# Report of Diagnostic Signs of Portal Phlebitis

**Published:** 1849-07

**Authors:** 


*Report of Diagnostic Signs of Portal Phlebitis. From extracts from the reports of the proceedings of the New York Pathological Society, selected and prepared by a Committee of Publication.*

Wm. C. Roberts, M. D., *Reporter.* (Submitted December 24d, 1845.)

The Committee appointed at the last meeting of the Society to present a report detailing the principal diagnostic signs of Phlebitis of the vena portæ, respectfully submit:

That in the subject committed to their consideration there are two elements: first, the symptoms common to phlebitis in general, and, second, those appertaining to the inflammation of the particular vein, and the organs connected with it, viz. the spleen, liver, and indirectly and occasionally only, the lungs.

Phlebitis is of two kinds; sub-acute or adhesive, i. e. leading to obliteration of the vessel; and acute, or that which is attended with the formation of pus within its cavity. In the first or sub-acute variety there is but slight general indisposition, but little local pain and tenderness, the vein soon becoming firm and hard, as if it contained solid matter; and dropsy of the parts beyond the trunk, commonly ensues. There is reason to believe, however, that a form of this variety may exist, attended with high and sthenic inflammatory action, sometimes, though not necessarily, or even generally fatal. Dropsy occurs rapidly, and may, or may not subside when the establishment of a collateral circulation ensues. In the acute or suppurative variety, the symptoms are much more severe. The attandant fever is of a typhoid character, depending on the admixture of pus with the circulating fluids, and its deposit in other frequently distant organs, viz: the liver, lungs, brain, kidneys, spleen, joints, serous cavities, and muscles, whereby new foci of irritation are created, and new pus poured into the general circulation. According to Schœnlein, the typhoid phenomena are peculiar to pus, and, as such, pathognomonic of the suppurative variety. Another peculiarity is the occnrrence of chills. They set in without regularity, so as to cause a continual intermittent fever, resembling an erratic intermittent. Several chills may occur per diem, sometimes as many days elapse without their occurring. In cases even where the veins of the liver are not inflamed, this organ, the lungs and the heart, nevertheless frequently take an important part in the characteristics of the disease. There is distension, and even pain of the right hypocondrium, bilious coated tongue, bitter taste in the mouth, nausea, and even vomiting; and even icterode color of the skin, conjunctiva and urine, which becomes brownish red, and often black. These phenomena are the more marked, the nearer the affected vein lies to the liver. The cardiac symptoms consist in signs of inflammation of the right side of the heart, violent pulsation below the ensiform cartilage, violent oppression, great restlessness, and frequent inclination to syncope. These signs are most marked in inflammation of the veins above the diaphragm. In addition to the above symptoms your committee may mention the occurrence of profuse sweats, and occasionally very unnatural and copious discharges from the bowels.

Your committee now proceed to examine whether symptomatologically, the cases reported as inflammation of the portal vein were really instances of phlebitis. They are six in number, viz. Drs. Graves, Reynaud, Lambron, and Moses, each one; and Dr. Schœnlein two cases ; of these, three were suppurative and three adhesive.

Of the cases of the suppurative variety. Lambron's patient at first had a natural pulse, and the tongue was white. In the course of a week it became dry, was loaded with a bluish crust, and the pulse had risen to 96. On the occurrence of pneumonia the pulse became hard and full; in two days after it began to sink, the pulse rising to 104, and being weak and compressible. In Schœnlein's first case there was at first violent fever, with burning heat of the skin, pulse 100, full and tense, urine dark and high colored. Tongue had a yellow coating ; in the course of the disease the fever moderated for a time, then assumed a hectic character, strength rapidly and remarkably succumbed. In his second case the tongue at first had a yellowish brown coating, there was much thirst, pulse varying from 100 to 110, skin burning hot. The fever quickly assumed the typhoid form, the pulse ran up to 128, was weak and compressible, the tongue became brown and dry, and strength failed rapidly. Such is a description of the *fever*, in the three cases of the suppurative variety, which have been presented to the Society, and, in the opinion of your committee, they show that, although at first of a sub-acute or acute sthenic character, it is speedily changed into the typhoid form, thus coinciding with the usual phenomena indicated as belonging to phlebitis in general.

Respecting the chills with suppurative variety : In Lambron's case, the patient, after suffering with some slight and obscure gastric symptoms, was seized with a shivering fit, which did not return under one week. Rigors then recurred frequently, easily simulating a regular intermittent fever. They then ceased to return at the end of another week In Schœnlein's first case, the patient was seized about the sixth day with a violent shaking chill, which was repeated at uncertain intervals;. on some days several occurred, and on one day as many as three: at one time they were absent for eighteen days. In his second case, chills did not set in till the fourth week, occurring at irregular intervals, sometimes two daily, and lasting more than an hour. She had only one interval of twenty-four hours freedom from chills, which then recurred, and continued daily up to her death, becoming more severe, so that some lasted for twelve hours, and recurred again after the lapse of scarcely six more. Here again we notice the characteristic phenomena of internal suppuration, which your committee would especially indicate to the society as a pathognomonic sign of the disease in question.

The third point of interest refers to the symptomatic evidences of a deposit of pus in adjacent or distant organs. In Lambron's case, about the twenty-second day, pain in the right side of the chest, cough, crepitant rale, and faint bronchial respiration were perceived. In Schœnlein's first case, no deposits took place in any organ. In the second, there was pain in the region of the spleen at an early date—the organ was found enlarged and tender about its hilus. Pain and tenderness existed about the liver. About the fifth week of the disease cough set in, pain in the chest, and circumscribed crepitant rattle was found in several places about either lung. Respiration quickly became oppressed, cough more severe, and sputa rusty and bloody. These two cases, in the opinion of your committee, while they exhibit no evidences of the cardiac affection mentioned by Schœnlein, a fact which may depend upon the non-residence of the pus conveyed into that organ, within its cavity, satisfactorily display the common attribute of phlebitis, namely, the developement in organs more or less distant from the seat of the phlebitis, of inflammation dependent upon the stasis within them of the products of the venus phlogosis. These stases are well known to the society under the name of metastatic abscess. The theory of the formation of these abscesses is so well explained by Dr. Budd, in his recent treatise on the Diseases of the Liver, that your committee have thought the paper worthy of being transcribed. "The globules of the purulent matter, mingled with the blood, are conveyed to the capillary vessels of the lungs, and it would seem, by becoming mechanically arrested there, excite each circumscribed inflammation and abscess. If any of the globules pass through the capillaries of the lungs, to the left side of the heart, they are sent in the arterial current to other organs, and, becoming arrested in the capillaries of these organs, create as in the lungs, inflammations of limited extent, rapidly passing on to abscess." "If the seat of the suppurative phlebitis happen to be one of the veins that go to form the vena portæ, the pus it contains will be carried first to the liver, before it be conveyed elsewhere, and then the abscesses will be found alone, or in greatest numbers in that organ."

The non-occurrence of metastatic abscesses in one of these three cases, shows that they are not to be expected in every case of phlebitis, and even when certain bilious symptoms do occur in particular cases as Schœnlein declares, they are not necessarily attributable to the presence of pus in the parenchyma of the liver, but form a characteristic phenomenon of the disease, independently of any local lesion, and are, therefore, of nature purely sympathetic. In proof of this, in Schœnlein's first case, in which there were no depots of of pus in the liver, the tongue constantly retained a yellow bilious coating, the urine was always dark brown, and impregnated with bile, the skin was at first yellowish, and became of a dirty green, and greenish, brownish, bilious and offensive matter was discharged from the stomach. The left lobe of the liver became swollen and tender. The value of this observation of Schœnlein's, however, in the opinion of your committee, is in this case diminished, by the consideration that it was one of inflammation of an hepatic vein, upon which circumstance the biliary phenomena may in part, if not wholly, have depended. All the three patients who were the subjects of the suppurative inflammation of an portal vein had profuse and viscid sweats; in Lambron's case there were passed dark green fiuid stools, Schoenlein's second case the stools were of a dark yellowish brown, mixed with much mucus. Schœnlein remarks that when the disease has lasted from four to six weeks, the stools often become black, tarlike, looking as if burnt, and often mixed with blood; and here again your committee would point out the close coincidence of the symptoms of these cases with those described as belonging to phlebitis in general.

Of the general symptoms of Portal Phlebitis. Of these but one, that seen by our fellow-member, Dr. Moses, was acute in its character, and as the patient was in a state of collapse during the three days that preceded her death, the symptoms indicated nothing pathognomonic of phlebitis; they were simply those of asthenia, but the previous history of the case, jaundice occurring after a *fit* of passion, fever, the rapid occurrence of œdema pedum, and ascites, *with the* enlargement of the superficial abdominal veins, might have suggested both the existence of obstructive disease of the vena portæ and cava, and that the closing symptoms in some degree depended upon venous inflammation, though obscured by the more immediate existence of a peritonitls. Your committee believe that the general symptoms are insufficient, in the commencement, to point out with certainty the existence of adhesive phlebitis : fortunately the fatal termination is neither certain nor speedy, and the progress of the case will, generally, ultimately lead to an an approximate diagnosis. Schœnlein, whose name stands high in Germany, and, indeed throughout Europe, as that of a most enlightened and accomplised practical physician, and to whom we certainly owe most of our knowledge of the disease which now occupies our attention, asserts, that the attending fever is of a peculiarly pungent, burning character, the causus of the ancients, and retains its inflammatory nature until the exudation of plastic lymph occurs within the vein. Then sudden distension, and swelling of the spleen sets in, because its blood cannot return back through the obstructed portal vein. Thus, in a few days, the spleen may reach the median line, and extend downwards, almost to the ilium. The enlargement of the spleen, even when not sufficient to appear beneath the ribs, may still be detected on careful percussion. Profuse hæmorrhages, also, are apt to occur from the bowels after the lapse of ten or twelve days, depending, also, as will readily be seen, upon obstruction of the trunk of the vena portæ. The blood may pass off in large quantities from the intestines, and all the signs of collapse from the loss of blood ensue. "The superficial veins of the abdomen also become enlarged and serpentine, in the endeavor to establish a collateral circulation;" facts remarkably exemplified in the more chronic cases of Stokes and Reynaud, and to some extent, in the acute case related by Dr. Moses. Your committee will take this opportunity of stating, that, in Dr. Watson's Lectures on the Practice of Physic, two cases of venous obstruction, attended with the development of the supplementary circulation on the superficies of the chest and abdomen, are related, and diagrams given illustrative of the fact.

Having pointed out the phlebitic nature of the cases of fatal venous inflammation, reviewed by your committee, they proceed to exhibit the local phenomena, in each variety, which may lead to the location of the disease in the particular vessel in question. These are, according to Schœnlein, pain in the middle point between the navel and ensiform cartilage, occurring spontaneously, occasionally becoming exceedingly severe, increased by pressure, and extending often along the tract of the veins of the spleen on the one side, which organ may be found tender, and along the tract of the vein towards the liver on the other side, which may also be be found puffy and tender, and, at times, extending even backwards toward the spine. The pain, in the adhesive variety, is dull and aching, and in the suppurative, severe, burning and gnawing. In Lambron's case, no mention is made of this central pain, but there was a constant pain in the right hypochondrium, with exacerbation, which the patient compared to smart cramps in the bowels. Jaundice is apt to occur at an early period: in Lambron's case which was sub-acute in its course, in the course of a few weeks: in Schœnlein's first case, in the course of a few days: in his second case, in a few hours, after a severe fit of anger and vexation, a mode of origin common to several cases of this kind, among others, that of Dr. Moses, in whom the joundice displayed itself also immediately. In the case mentioned by Andral, in his clinique, the patient, after laboring for some time under symptoms of fever and gastroenteritis, was attacked with pain and tension in the region of the liver, followed by jaundice. Reynaud's patient labored for more than twelve months under jaudice, accompanied by wasting of the flesh and prostration of strength. He had constant pain in the epigastrium and swelling of the feet; symptoms which as Stokes remarks, would have generally been regarded as those of chronic hepatitis. To these evidences of hepatic disorders may be added the frequent presence of bitter taste, a yellow coated tongue, loss of appetite, hiccup, nausea, and vomiting, more or less vitiated bilious discharges from the bowels, and the presence of the coloring matter of the bile in the urine; pain, tenderness, and in some cases, swelling in the region of the liver. In the patient seen by Dr. Moses, after the lapse of several months the skin still retained an icterode hue, the excretions were dark green and bilious.

Obstruction of the vena portæ leads almost necessarily to abdominal dropsy, It existed in the cases seen by Moses and Reynaud, both of which were cases of the adhesive variety. In those of the suppurative variety, reported by Schœnlein and Lambron, this did not occur, a fact which is of importance in the distinctive diagnosis of these two forms of phlebitis. We may remark in passing, that in Reynaud's case, there existed emaciatien coupled with canine appetite, and Graves, in cases supposed to be of this kind, not verified, however, by post mortem examination, has observed the same thing; leading to the interference that the mesenteric and other abdominal veins take part in the process of absorption of the nutritive elements of the food from the intestines.

Such are the evidences which your committee have to exhibit, leading to the inference that these and all other similar cases of phlebitis, are in reality cases in which the veins of the liver are the seats of the particular lesion. In those cases (Reynaud's) in which anasarca has co-existed with the other symptoms, the cause has been found in the extension of the inflammation to the vena cava. Your committee feel bound to remark that, whereas, these prominent bilious symptoms attend upon the disease at its acme, some may be absent at its commencement: thus the tongue may be at first white, and only become yellow at a later period, and the bilious diarrhœa is usually preceded by constipation. The ages of the patients in four cases are mentioned. Lambron's patient was sixty-nine years of age, Dr. Moses's thirty two, Schœnlein's thirty-three and twenty-six respectively. Of the five cases, in which the sex is given, three were males and two females.

The more important results, developed by the preceding report, your committee have endeavored to embody in the following corollaries:

1st. Phlebitis is of two kinds—acute, sub-acute or adhesive, acute and suppurative. In the first variety, generally, the symptoms, general and local, are of less intensity; in the second, they are much more severe, the fever is of a typhoid form, only, according to Schœnlein, met with in this variety, of which it is therefore pathognomonic.

2d. That, according to the same authority, the local symptoms are the same in both, but the febrile reaction and mode of death are different, and that in the adhesive form the early diagnosis is difficult, if not impossible, and, at a later stage, only approximative.

3d. That chills always and only occur in the suppurative variety, causing the remittent form of fever, which is the natural type of the disease, to resemble an irregular or erratic intermittent, and are markedly indicative of the true nature of the case.

4th. That the six cases examined by your committee were instances of phlebitis, shown by their accordance with the general symptoms of the disease, not less than by the post mortem examinations, three being of the adhesive, and three of the suppurative variety.

5th. That in the suppurative variety, symptoms of hepatic, splenic, pneumonic and cardiac diseases display themselves, which depend upon the deposit of pus by metastasis in the parenchymatous viscera; but this does not invariably happen, nor always depend upon the presence of transported pus when it does.

6th. That bilious phenomena may occur in general phlebitis, from this cause, without an inflammation of the veins of the liver necessarily existing; or that they may depend solely upon the latter cause, in the absence of purulent deposits in the hepatic parenchyma.

7th. That certain symptoms, as pain in the middle point between the navel and ensiform cartilage increased by pressure, extending along the tract of the splenic vein, and those of the liver, differing in each variety, or in the right hypochondrium only, jaundice, frequently a primary symptom, and resulting after fits of passion, a bitter taste in the mouth, a yellow coated tongue, vitiated bilious discharges, a bilious tinge of the urine, and dropsy in the adhesive variety only, are the local signs which point to the existence of phlebitis in some hepatic vein, although, for reasons previously assigned, these are not in every case characteristic nor uniformly present in the commencement of every case.

8th. That it is only in cases where plastic lymph is effused into the vein, that the distension of the superficial abdominal veins occur; and that neither this, nor the enlargement of the spleen with which it is so often associated are pathognomonic of portal phlebitis, but may depend upon obliteration of other large abdominal venous trunks.

9th. That the fever of phlebitis, in general, is, in both varieties, of the burning pungent kind; in the adhesive, retaining its inflammatory character until the exudation of plastic lymph occurs; in the suppurative, soon assuming the torpid typhoid character, to which variety, also, the chills and metastatic abscess are peculiar.

10th. And lastly, that profuse and viscid sweats are among the distinctive evidences of suppurative phlebitis.—*New York Journal of Medicine.*

